# Addition of Broad Bean Hull to Wheat Flour for the Development of High-Fiber Bread: Effects on Physical and Nutritional Properties

**DOI:** 10.3390/foods9091192

**Published:** 2020-08-28

**Authors:** Qianqian Ni, Viren Ranawana, Helen E. Hayes, Nicholas J. Hayward, David Stead, Vassilios Raikos

**Affiliations:** Rowett Institute, University of Aberdeen, Aberdeen AB25 2ZD, UK; q.ni.18@abdn.ac.uk (Q.N.); vranawana@abdn.ac.uk (V.R.); h.hayes@abdn.ac.uk (H.E.H.); n.j.hayward@abdn.ac.uk (N.J.H.); d.stead@abdn.ac.uk (D.S.)

**Keywords:** broad bean hull, *Vicia faba*, bread, gluten, high fiber, texture

## Abstract

The seed coat (hull) of broad bean (*Vicia faba*) (BBH) is a significant secondary product of processing with a promising nutritional profile. Bean hull has a high fiber content (49%), yet it remains underexploited as an ingredient by the food industry. This study investigated the potential of this secondary product to partially replace wheat flour for the development of high-fiber breads. Bread formulations with a range of supplementation levels (0%, 11%, 21% and 31%) were developed and tested for their nutritional and physical properties. The proximate composition of breads revealed that at 31% replacement, the fiber content was 19.19 g/100 g bread, which was significantly higher (*p* < 0.05) than control breads (3.62 g/100 g bread). The physical (specific volume, density and color) and textural properties of breads were affected by the addition of bean hull. Specific volume and hardness of breads were significantly reduced at ≥21% replacement compared to the control, which may reduce acceptability of the product by some consumer groups. Enzyme-linked immunosorbent assay (ELISA) showed that the gluten content of breads was significantly reduced with bean hull addition (62% depletion for 31% replacement). At 11%, 21% and 31% replacement, one portion (80 g of bread) contains 6.8 g, 11.6 g and 15.3 g of dietary fiber, respectively, which contributes 23%, 38% and 51% of the recommended daily fiber intake (30 g/day). In conclusion, bean hull can be a valuable source of dietary fiber in bread formulations. The study showed BBH could be used to replace up to 21% of the wheat flour without significantly impacting on bread texture and volume.

## 1. Introduction

Bread is an important staple food worldwide, which is typically made from wheat (*Triticum aestivum*) flour, salt, sugar, water, fat and baker’s yeast. Wheat flour is a key ingredient in bread making due to its unique endosperm protein composition (gliadins and glutenins). The latter are responsible for the formation of “gluten”, which is the term commonly used to describe the viscoelastic network of the dough that allows the entrapment of CO_2_ and contributes to the desired bread texture and volume [1]. Food products made of wheat contribute significantly towards meeting dietary energy and protein intakes, and therefore their moderate consumption (250–350 g of bread daily) is recommended by nutritional bodies [2]. The majority of wheat flour used for bread making is refined due to consumer demands. However, the refinement process diminishes nutritional quality, particularly in its fiber, vitamin and phytochemical contents [3]. To compensate for these nutritional losses, a multi-grain approach is often adopted by the baking industry. This approach uses refined wheat flour in combination with nutrient-dense flours from other crops to improve the nutritional profile of the end-product and meet consumer demands for healthier food products [4].

The important role of dietary fiber (DF) in disease prevention has become increasingly apparent in recent years. Its beneficial effects on human health include reduced risk of coronary heart disease, diabetes and colon cancer [5,6], and are attributed to the physicochemical and functional properties of fiber. The daily recommendations for DF (30 g/day) are not met by the majority of the adult population in westernized countries. Thus, the incorporation of DF into frequently consumed processed foods could be an effective approach for meeting recommendations and satisfying consumer demands for more nutritious food products.

Broad bean (*Vicia faba*; field bean, horse bean, fava bean) is a legume belonging to the family Fabaceae and forms an important part of diets in many parts of the world, notably in Middle Eastern, Mediterranean, Asian, South American and African regions. As a leguminous crop it is often used in crop rotation for soil enrichment. The principle dietary element of the plant is the seed, which is consumed fresh and dried, and similar to other legumes has a favorable nutritional profile rich in protein, fiber, micronutrients and phytochemicals [7]. The processing of combinable broad beans involves the removal of the seed testa (hull) constituting approximately 12–14% of seed weight, as this is believed to improve eating quality and appearance. Therefore, the hull is a significant secondary product of bean processing which is largely underutilized. Previous research indicates dehulling significantly reduces the total non-starch polysaccharide (NSP) content in the coarse fractions of broad beans, thus affecting the dietary fiber content in the legume post-processing [8].

Little work has been carried out to assess the potential of secondary products derived from broad bean processing as a human food despite it being a food grade material that is rich in fiber and phytochemicals. Previous work shows that fiber from broad bean pods can be used to enhance dough development and to improve the textural profile of bread [9]. Data gathered by our group over the last two years show that broad bean hull has a favorable nutritional profile compared to comparable material such as wheat bran. The aim of this study was to assess the potential of using broad bean hull (BBH) for developing high-fiber breads. The effect of white flour partial replacement with BBH on bread quality (volume, texture, color) and nutritional composition was investigated.

## 2. Materials and Methods

### 2.1. Materials

White flour (Tesco Stores Ltd., Welwyn Garden City, UK), yeast (Allinson’s Flour, Peterborough, UK), Trex vegetable fat (Princes Limited, Liverpool, UK) and Saxa fine sea salt (Premier Foods, Cheshire, UK) were purchased from a local supermarket (Tesco, Aberdeen, UK). Broad bean hull (variety ‘Fuego’) was kindly provided by Askew and Barrett (Pulses) Ltd. (Cambs, UK). The hull was obtained through a standard legume processing methodology in a commercial large-scale plant. Briefly, following sampling and inspection dry broad beans were destoned and defected seeds and foreign material removed. The beans were colour sorted and dehulled using a Berhard Keller abrasion dehuller. The resulting hull was sieved to remove excess cotyledon particles, and then milled to a particle size of below 0.5 mm using a rotor mill fitted with a 0.5 mm sieve (ZM 20, Retsch, Haan Germany) at the James Hutton Institute (Dundee, UK). All reagents used were of analytical grade.

### 2.2. Bread Preparation

Control breads was prepared using 100% wheat flour. All dry ingredients (including salt) were combined with water manually until a cohesive dough was formed and rested for ten min before kneading for five min by hand. Treatment breads were made by replacing 11%, 21% and 31% of the white flour with BBH and the water content and salt were adjusted accordingly (Table 1). After kneading, the dough was covered with a damp cloth and proofed for 90 min at room temperature. After the first proofing, the dough was divided into two (each dough weighed 175 g), weighed and proofed again for 60 min at room temperature. The rolls were baked in a pre-heated fan-assisted oven at 200 °C for 23 min, cooled at room temperature for 1.5 h and their weights recorded before storing at 4 °C overnight prior to physical and nutritional analysis.

### 2.3. Physical Properties

#### 2.3.1. Specific Volume, Density and Weight Loss

Bread volume was determined using a modified standard rapeseed displacement method 10–05 (American Association of Cereal Chemists (AACC) International, 2000) [10], in which sesame seeds were used instead of rapeseed. The loaf specific volume was calculated as loaf volume divided by loaf weight (1.5 h after baking). Density was calculated as loaf weight divided by loaf volume. Weight loss was computed as below:

$$\mathrm{Weight}\;\mathrm{loss}\;(\%)=\frac{\left(\mathrm{initial}\;\mathrm{weight}\;\mathrm{before}\;\mathrm{baking}\;-\;\mathrm{loaf}\;\mathrm{weight}\;1.5\;h\;\mathrm{after}\;\mathrm{baking}\right)\times 100}{\mathrm{initial}\;\mathrm{weight}\;\mathrm{before}\;\mathrm{baking}}$$

#### 2.3.2. Bread Crust and Crumb Color

Crust and crumb color were measured by a Konica Minolta CR1 10 color meter (Konica Minolta Solutions Ltd., Basildon, UK). Breads were sliced using a Swan food slicer (model SFS102, adjusted to 15 mm thickness), crust color measured at the middle point of the top crust of bread, and crumb color measured at the middle point of the central slice in triplicate under controlled lighting. Measurements were made using the International Commission on Illumination (CIE) L* (lightness), a* (redness to bluishness), b* (yellowness to greenness) system. Brownness index (*BI*) and whiteness index (*WI*) were calculated as follows [11]:

(1) $$BI=\frac{\left(100\left(X-0.31\right)\right)}{0.17}$$

where, $X=\frac{\left(a*\;+\;1.75L*\right)}{\left(5.645L*\;+\;a*\;-\;3.012b*\right)}$.

(2) $$WI=100-{\left({\left(100-L*\right)}^{2}+\;a{*}^{2}+b{*}^{2}\right)}^{1/2}$$

### 2.4. Texture Analysis

For each bread, the middle slice and the two slices on either side of it were used for texture profile analysis (TPA) 24 h after baking using a CT3 Texture Analyser (Brookfield Engineering Laboratories, Inc., Middleboro, MA, USA) fitted with a cylinder probe (TA11/1000, diameter = 25.4 mm). Data was recorded using Texture Proc CT V1.3 Build 15 software (Brookfield Engineering Laboratories Inc.). All measurements were made in duplicate at a pre-test speed of 2 mm/s, test speed of 1 mm/s, trigger force of 10 g, and 50% compression [9]. The texture properties determined were hardness, resilience, adhesiveness, cohesiveness, springiness and chewiness.

### 2.5. Scanning Electron Microscopy (SEM)

Bread samples were freeze dried and manually fractured into cubes using the tip of a razor blade. Samples were made electrically conductive by coating with a thin layer of gold-palladium using a Quorum Q150 ES sputter coater (Quorum Technologies Ltd., East Sussex, UK). BBH sample was sprinkled on to the stub used to display the sample and then treated with the gold. The samples were imaged at an accelerating voltage of 10 kV using a Zeiss EVO MA10 SEM (Carl Zeiss Ltd., Cambridge, UK). SEM pictures of the newly exposed surface of BBH were taken at 200× and 1000× magnifications and those of bread samples were taken at 1000× and 3000×.

### 2.6. Nutritional Analysis

#### 2.6.1. Proximate Composition Analysis of BBH Powder and Breads

The proximate composition of BBH powder and breads was analysed in duplicate using standard Association of Official Analytical Chemists (AOAC) analytical procedures [12]. Dietary fiber (DF) was determined by general method AOAC 991.43, which is the enzymatic-gravimetric method most used in determining the DF content of foods [13]. Non-starch polysaccharides (NSP) were determined by the Englyst method [14]. These analyses were conducted by a United Kingdom Accreditation Service (UKAS) certified testing facility (Huson and Hardwick’s Food Test Lab, Merseyside, England) and accredited to ISO 17025:2017.

#### 2.6.2. Gluten Quantification

The gluten content of each sample was analysed using the AgraQuant^®^ Gluten G12^®^ Assay (AACC International Method 38–52.01) (Romer Labs UK Ltd., Runcorn, Cheshire, UK). Samples were extracted in duplicate according to the manufacturer’s standard method. Briefly, samples were blended by a blender (Essex Scientific Laboratory Supplies Ltd., Essex, UK) for about five min and 0.25 g weights were mixed with 2.5 mL of extraction solution before incubating at 50 °C for 40 min. This was followed by the addition of 7.5 mL of 80% ethanol and agitation for 60 min at room temperature, and centrifuged at 2000× *g* for ten min. The clear aqueous layer between the particulate sediment and supernatant was obtained and stored at 4 °C until analysis. The extracted bread samples were diluted to 1:5000 and 1:10,000 with diluent buffer and loaded on to antibody coated microwells in duplicate along with gluten standards (0, 4, 20, 80 and 200 ppm) before analysis.

### 2.7. Statistical Analysis

Statistical analysis was performed by SPSS software version 25.0 (SPSS for Windows 22, SPSS Inc., Chicago, IL, USA). Data on the physical properties, texture analysis, proximate analysis and gluten content were analyzed by one-way ANOVA and using post-hoc Tukey tests where significant model effects were observed. Statistical significance was defined as *p* < 0.05, and results expressed as means and standard deviations (SD).

## 3. Results and Discussion

### 3.1. Physical Properties

#### 3.1.1. Specific Volume, Density and Weight Loss

**Figure 1:**
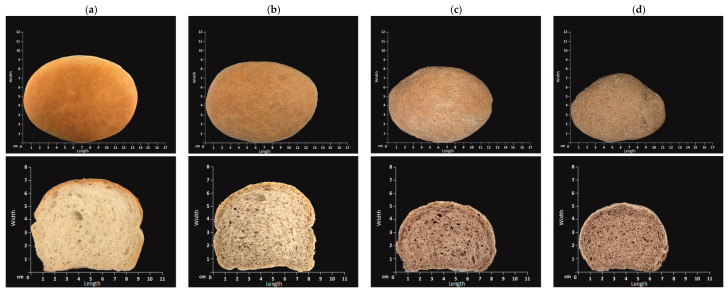
Loaves and slices of bread samples: (**a**) control; (**b**) 11% BBH bread; (**c**) 21% BBH bread; (**d**) 31% BBH bread.

All bread loaves were made using a standard bread making process which included mixing, fermentation and baking. The visual appearances and dimensions of all bread loaves and slices are shown in Figure 1. The 31% BBH bread loaf size was the smallest whilst the control bread was the largest. The control bread slice and 11% BBH bread slice presented similar visual appearance. When the fortification level was higher than 11%, the crumb surface was darker and presented smaller porous structure compared with the control bread.

The results of specific volume, density and weight loss are shown in Table 2. The specific volume is the most important parameter in bread making, which indicates final gas retention in the bread and affects consumer preference. The control bread had the greatest specific volume (4.11 ± 0.08 mL/g). The specific volumes of BBH fortified breads varied from 1.65 to 3.65 mL/g and were significantly lower compared to the control (*p* < 0.05). This loss of volume can be attributed to the dilution of gluten content and the increase in fiber content of BBH. Fiber particles can impede proper gluten development by cutting through gluten strands, thus inhibiting the formation of a viscoelastic network, which results in weakening of the dough [15,16]. A weakened gluten network during dough formation can hinder rising of the bread which results in decreased loaf volume.

Breads at 31% fortification level had the highest density (0.61 ± 0.02 g/mL) whereas the control bread was the least dense (0.24 ± 0.004 g/mL), and the density was significantly increased while increasing the BBH fortification level (*p* < 0.05). Previous research has shown that fiber can modify food structure and increase its density because of its fibrous nature [17]. Additionally, the highest weight loss was observed in the control breads (13.79 ± 0.59%), while it decreased gradually with addition of BBH, and only significant differences among control, 21% and 31%, as well as between 11% and 31% breads were observed (*p* < 0.05). This is possibly because fiber is rich in non-starch polysaccharides (NPS’s) which can bind to water through the formation of hydrogen bonds [18]. Fiber tends to absorb water which limits water availability for other key ingredients in gluten development such as gluten and starch. There are two main effects of fiber on bread structure: it allows more water to be absorbed within the bread structure and alters dough development [19]. This latter is manifested by prolonged mixing time, longer dough development time and reduced extensibility in fiber-rich dough [20]. Previous research revealed that pan bread fortified with rice bran had lower baking loss [21]. The authors suggest that the high fiber content of rice bran induced water interactions through the formation of hydrogen bonds resulting in lower baking losses.

BBH has a similar nutritional profile to wheat bran but higher DF content, particularly in insoluble fiber [22]. Table 3 shows that BBH is low in fat and carbohydrate content and contains approximately twice the amount of fiber compared to wheat bran (~40 g/100 g) [23]. Furthermore, acid hydrolysis of the NSP revealed that the main monomeric sugars are glucose, xylose and uronic acid. It was reported that wheat bran fiber had a negative effect on the specific volume of bread as it produced less viscous dough which could not retain entrapped air bubbles during baking [24]. Adverse effects on dough properties due to the addition of bran to flour tend to increase with higher levels of wheat flour substitution with bran [25]. Numerous studies have shown a decrease in loaf volume after the addition of fibers from agricultural secondary products such as mango peels, pineapple pomace, apricot kernels and hazelnut testa [11,24,25,26]. In the current study, apart from lowering gluten content, BBH may also interfere with gluten structure resulting in reduced gas retention. Besides, it has been suggested that fiber can retain water during breadmaking and make it less available for development of the starch-gluten network [27].

#### 3.1.2. Bread Crust and Crumb Color

The results of bread crust and crumb color are shown in Table 2. Color is one of the most important parameters to determine bread quality and acceptability and is influenced by the Maillard reaction and caramelization during baking. The former involves reactions between reducing sugars and the free amino acid side chain of proteins which results in the formation of brown pigments [25]. The latter is a non-enzymatic reaction of sugars under high temperature [27].

In the current study, the lightness (L*) of the crust color significantly increased at fortification levels 11% and 21% but decreased at 31%. A similar trend was observed for the WI values of all fortified breads compared with the control bread. The BI of the control bread was the highest and then significantly dropped when the fortification level was above 11%. There was no significant difference for BI between 21% and 31% breads. Although BBH imparted a brown color on the surface of the bread, the BI measured was significantly lower when compared with the control bread. One possible explanation for this may be associated with the decreased total sugar and protein content in breads fortified with BBH. Since the overall content of substrates for the Maillard reactions and caramelization is reduced, less brown pigment formation is expected.

Regarding crumb color, lightness was significantly decreased when BBH fortification was above 11%. There was a significant decrease in WI for the BBH fortified breads. No significant difference was noted between the control and the 11% BBH bread but for samples at higher fortification levels (≥21%), BI was significantly increased. However, the difference of BI was not significant between 21% and 31% breads. Since the temperature during baking is not above 100 °C inside the bread [28], the crumb color is determined by the colors of ingredients used to make the recipe [25]. These results indicate that incorporation of BBH into bread has darkening effects in the inner part of bread, which is attributed to the color effects imparted by the ingredients. Some studies also reported the darker crumb color of baking products after addition of DF enriched plant by-products [11,21,25].

### 3.2. Texture Analysis

Texture properties of all bread samples are presented in Table 4. Hardness is the most important parameter which reflects bread quality. Compared with the control, BBH supplementation increased the hardness of breads, and this effect was significant (*p* < 0.05) for high levels of fortification (31%). Similar results have been reported showing that the addition of plant-based secondary products rich in DF increased the hardness of breads [11,24,25]. This effect is attributed to the thickening of the walls surrounding the air cells in the crumb and is also associated with the formation of a cross-linked network between gluten proteins and starch [25]. Furthermore, DF interacts with gluten proteins and these interactions may be unfavorable for the formation of a viscoelastic dough [29].

Cohesiveness relates to the internal resistance of crumb structure and the ability of the crumb to deform before it fractures. Bread with low cohesiveness is susceptible to rupture [10]. There was a significant reduction of cohesiveness in BBH fortified breads compared to control breads. The 11% BBH bread had the highest cohesiveness (0.47 ± 0.05) among all fortified breads. A high cohesiveness indicates an ability to form a bolus instead of crumbling during mastication. The reduction of cohesiveness in BBH fortified breads indicates they are more prone to disintegration during masticating.

Springiness is a measure of how much the crumb springs back after the first compression [30]. Chewiness is derived from springiness, hardness and cohesiveness and reflects the energy required to masticate food to a ready-to-swallow state [31]. Compared with control breads, 11% bread had similar springiness, but it significantly decreased at 21% and 31% BBH breads (*p* < 0.05). Chewiness of breads significantly increased when the supplementation level was above 21% and there was no difference between 21% and 31% BBH breads. Other research has also found that the addition of DF (wheat bran) could significantly increase chewiness but had no significant effect on springiness [21]. This is possibly because only 10% of wheat bran was used in their bread formulation, which contained less insoluble fiber compared with the present study.

By definition adhesiveness is the requirement of a force to remove breadcrumbs that adhere to the palate [21]. A significant increase of adhesiveness was observed between the control bread and all fortified breads. Adhesiveness and springiness can be used to indicate the elasticity of breads. This characteristic of breads is related to the interaction among gluten proteins, starch and water during breadmaking. This interaction was altered with the addition of DF as mentioned before and hence changed the texture of breads.

Resilience indicates the speed of crumb recovery after compression [32]. There was a significant decrease of resilience with increasing BBH in breads. The texture profile of bread samples indicates that the addition of BBH can affect their sensory properties by altering texture. This is likely to be due to the formation of a weakened gluten network structure, with adverse effects on texture observed at 31%.

### 3.3. SEM

**Figure 2:**
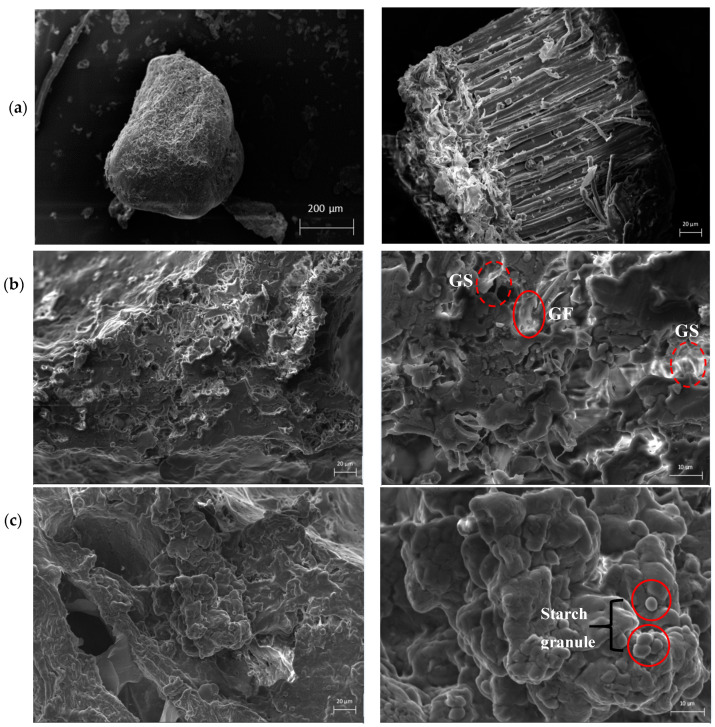
Microstructure of BBH, control breads and 21% BBH fortified bread. Scanning Electron Microscopy (SEM) was carried out at 200× and 1000× magnification in (**a**) BBH; 1000× and 3000× magnification in (**b**) control bread and (**c**) 21% BBH bread. GS, gluten strand; GF, gluten film.

SEM was used to study the microstructure of the BBH, control bread and 21% BBH bread. The micrographs of BBH (Figure 2a) show the fiber structure in detail. Under 200× magnification, an irregularly shaped particle can be observed, which is similar in morphology to pea fiber microstructure [33]. When magnified to 1000×, BBH presented as a polymeric network of densely packed elongated and fibrillar structures.

Control bread (Figure 2b) had a rougher surface of crumb microstructure compared to the 21% BBH bread (Figure 2c) in 1000× magnification. Under 3000× magnification, control bread showed a rough layer with more gluten strands while 21% BBH bread had a more continuous and smoother layer. Besides, 21% bread tended to show naked starch granules whereas control bread had gluten films and appeared to be covered with intact starch granules. Those microstructure images support the hypothesis that there are interactions among gluten, starch and dietary fiber from BBH. Furthermore, the dilution of gluten proteins by starch may produce a weakened gluten network of reduced strength. The existence of increased starch granules can also produce an increasing number of gas cells, often surrounded by amorphous structures. Similar microstructure as the 21% BBH bread was observed in a high-protein hybrid bread which was formulated with broad bean, carob flour and psyllium [34]. Authors also suggested that the structure of the supplemented bread further supported the theory of a network among gluten, non-wheat proteins and fiber. Additionally, more naked starch granules were observed in a reduced-gliadin wheat dough microstructure [2].

### 3.4. Nutritional Properties

#### 3.4.1. Proximate Composition Analysis

The results of the proximate composition of bread samples is shown in Table 5. Supplementation significantly decreased the energy content of the BBH bread samples compared to the control. The moisture content was significantly increased when the supplementation level was above 11% and reached the highest value at 21%. There was no significant difference of moisture content between 11% and 21%. This result is attributed to moisture loss during baking which agrees with the weight loss data shown in Table 3.

There was a significant decreasing trend in proteins and available carbohydrates of fortified breads compared to the control. Regarding total sugar content, there was a reduction in fortified breads compared to the control with the highest reduction seen at 31%. These reductions are indicative of a less extended caramelization process and Maillard reactions, therefore, resulting in decreased crust browning in the fortified breads.

The total fat content of fortified breads ranged from 2.81 to 3.60 g/100 g. The total fat content was significantly decreased above 11% fortification compared to the control. A significant reduction in saturated and monounsaturated fat content was observed in fortified breads compared to the control bread. There was also a decreasing trend in polyunsaturated fat contents in fortified breads but no significant difference between 11% and 21%.

There was a non-incremental effect on sodium content of bread samples. Compared with the control, sodium was significantly higher in 11% and 31% fortification levels but lower in the 21% treatment. This variable result could be attributed to the uneven blending of dry ingredients during breadmaking. The ash content of bread samples ranged from 1.74 to 2.10 g/100 g with the 31% treatment having the highest value. This indicates that BBH contains minerals which may attribute to the increase in the ash content.

The total DF content of fortified breads ranged from 8.53 to 19.19 g/100 g corresponding to an increase in fiber of up to fivefold from the control value. The increase of DF was statistically significant among bread samples. In the UK, the daily recommended DF intake for adults is 30 g/day [35]. To contribute to this by consuming one portion of bread (80 g), 11%, 21%, 31% replacement contribute 23%, 38% and 51% of the daily intake, respectively. Notably, two portions of 31% bread can provide the daily intake of DF, not necessarily at one eating occasion. Previous studies have shown that at similar fortification levels (30% replacement of white flour with coarse or fine wheat bran), the total DF reached its highest value of 18.47 g/100 g bread [36]. This means that BBH addition can be used similarly to wheat bran for bread fortification purposes.

The proximate composition of bread samples showed that the addition of BBH provides better nutritional quality with notably increased DF content. Similar effects were revealed when bakery products were fortified with other crops such as rye flour, rice bran and pineapple fiber [21,24,37].

#### 3.4.2. Gluten Quantification

The total gluten content, determined by G12 competitive ELISA assay, is expressed in parts per million (ppm) per loaf and milligram (mg) per 50 g serving size of bread. The G12 monoclonal antibody can specifically recognize the QPQLPY present in a toxic 33-mer peptide, which is the main immunodominant toxic peptide that causes celiac disease (CD) [2]. The G12 antibody was useful in detecting gluten-relevant peptides due to its sensitivity and epitope preferences, thus reducing the potential toxicity of food for patients with CD [38].

Although gluten content was still high after the addition of BBH, there was a reducing trend with increasing fortification levels. There was no significant difference in gluten content between control bread and 11% BBH breads, but a significant reduction occurred when the fortification level was above 21% (Table 6). The 31% BBH bread showed the lowest gluten content (7259.00 ± 748.00 ppm per loaf; 2335.00 ± 240.51 mg/50 g serving size). Compared with the control, gluten depletion was calculated to be 8%, 31% and 62% per loaf for breads with 11%, 21% and 31% BBH, respectively. The observed reduction of gluten at high fortification levels (31%), cannot be attributed only to the dilution of gluten content with BBF addition, but there may be an additional effect. Increasing by 10% the BBH in the bread recipe (from 21% to 31%), the amount of gluten depleted doubled (from 31% to 62%). This effect may be ascribed to interactions between BBH DF and gluten proteins, which limited gluten availability. A crucial step during bread making is the crosslinking of gluten proteins with disulfide bonds to ensure the formation of a viscous dough. Dietary fiber could disrupt the formation of intermolecular disulphide bonds between gluten molecules by forming protein-polysaccharide complexes, thereby decreasing gluten protein cross-linking [39].

## 4. Conclusions

Overall, this proof of concept study showed that BBH could be effectively used to produce high-fiber breads and lower gluten contents. Among the breads supplemented with BBH (11%, 21%, 31%), 11% fortification had minimum adverse effects on physical properties and texture profiles. On the other hand, fortification at 31% produced breads with the most desired nutritional profile. Approximately two slices of BBH-containing bread (156 g) at 31% replacement of white flour contain 30 g of DF, which corresponds to the recommended daily DF intake. However, based on the findings of this study, the amount of BBH used to replace white flour in the recipe needs to be lower than 21% to compensate for significant adverse effects on bread texture and volume. Additional work is needed to elucidate the interactions among the dough components including wheat proteins and fiber polysaccharides, and to better understand the effect of BBH on the rheological and sensory properties of the BBH-fortified dough, in order to optimize bread recipes and improve quality attributes.

## Figures and Tables

**Table 1:** Formulations for control and broad bean hull (BBH) enriched breads.

Ingredient	Control	11%	21%	31%	% of Flour
	Quantity (g)				
Strong white wheat flour	75	75	75	75	
BBH	-	9	20	34	-
Yeast	2	2	2	2	3
Salt	1.4	1.7	1.8	2.2	2
Fat	3.75	3.75	3.75	3.75	5
Water (lukewarm)	59	66	75	86	78.9

The weights of the above ingredients were tripled for control, 11% and 21%, and those of 31% were doubled to obtain two equivalent doughs of 175 g. The percentages given for salt and water account for total dry powder (% dry powder).

**Table 2:** Physical characteristics of control bread, 11%, 21% and 31% of BBH fortified breads.

Bread Type	Specific Volume (ml/g)	Density (g/mL)	Weight Loss (%)	Colour					
				Crust			Crumb		
				L*	WI	BI	L*	WI	BI
Control	4.11 ± 0.08 ^a^	0.24 ± 0.004 ^a^	13.79 ± 0.59 ^a^	51.90 ± 2.12 ^a^	40.07 ± 1.71 ^a^	113.52 ± 6.13 ^a^	62.65 ± 1.18 ^a^	59.91 ± 1.07 ^a^	26.75 ± 0.69 ^a^
11%	3.65 ± 0.07 ^b^	0.27 ± 0.01 ^b^	12.83 ± 0.01 ^a^^b^	54.77 ± 0.64 ^b^	45.89 ± 0.54 ^b^	82.63 ± 2.88 ^b^	54.17 ± 1.47 ^b^	52.15 ± 1.32 ^b^	33.33 ± 0.52 ^a^
21%	2.30 ± 0.03 ^c^	0.44 ± 0.01 ^c^	11.61 ± 0.51 ^b^^c^	55.25 ± 0.77 ^b^	51.29 ± 1.03 ^c^	48.15 ± 3.22 ^c^	35.55 ± 4.40 ^c^	34.25 ± 4.33 ^c^	51.74 ± 8.46 ^b^
31%	1.65 ± 0.05 ^d^	0.61 ± 0.02 ^d^	11.17 ± 0.91 ^c^	43.47 ± 1.32 ^c^	41.56 ± 1.22 ^a^	46.88 ± 1.06 ^c^	40.67 ± 1.50 ^d^	38.89 ± 1.44 ^d^	49.83 ± 2.46 ^b^

Data are expressed as mean ± SD. WI is whiteness index: 100 − [(100 − L*) ² + a*² + b*²]^½^; BI is brownness index: [100 × (X − 0.31)]/0.17, where X = (a* + 1.75 L*)/(5.645 L*+ a* − 3.012 b*) (Data for a* and b* are not shown). Different letters within each column represent significant differences (*p* < 0.05) in physical properties.

**Table 3:** Proximate composition of BBH powder.

Content		Broad Bean Hull
Energy (kcal)		192
Ash		2.5
Moisture		9.0
Protein		5.3
Fat		0.4
Carbohydrate		1.1
Fiber		81.7
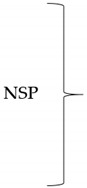	Rhamnose	0.4
	Fucose	0.1
	Arabinose	0.9
	Xylose	10.0
	Mannose	0.1
	Galactose	0.8
	Glucose	11.6
	Uronic acid	8.5

All values are per 100 g of sample. Unless otherwise stated units are in g.

**Table 4:** Texture properties of control bread, and 11%, 21% and 31% of BBH fortified breads.

Bread Type	Hardness (g)	Resilience	Adhesiveness (mJ)	Cohesiveness	Springiness (mm)	Chewiness (mJ)
Control	558.67 ± 37.19 ^a^	0.28 ± 0.02 ^a^	0.47 ± 0.34 ^a^	0.54 ± 0.02 ^a^	7.19 ± 0.20 ^a^	21.17 ± 1.62 ^a^
11%	619.83 ± 35.40 ^a^	0.22 ± 0.02 ^b^	1.07 ± 0.73 ^a^^b^	0.47 ± 0.05 ^b^	7.32 ± 0.18 ^a^	21.08 ± 3.21 ^a^
21%	2100.83 ± 344.69 ^b^	0.13 ± 0.01 ^c^	1.18 ± 0.89 ^a^^b^	0.37 ± 0.02 ^c^	6.60 ± 0.22 ^b^	50.53 ± 7.43 ^b^
31%	4666.17 ± 215.03 ^c^	0.06 ± 0.01 ^d^	1.70 ± 0.80 ^b^	0.21 ± 0.01 ^d^	5.22 ± 0.42 ^c^	50.68 ± 3.84 ^b^

Data are expressed as mean ± SD. Means with different letters within each column are significantly different (*p* < 0.05).

**Table 5:** Proximate composition of control bread, and 11%, 21% and 31% of BBH fortified breads.

Bread Type	Energy		Protein (g/100 g)	Available Carbohydrates (g/100 g)	Total Sugars (g/100 g)	Fat (g/100 g)				Dietary Fibre (g/100 g)	Sodium (mg/100 g)	Moisture (g/100 g)	Ash (g/100 g)
	Kcal/100 g	kJ/100 g				Total Fat	Saturates	Mono-Unsaturates	Poly-Unsaturates				
Control	269 ± 0.71 ^a^	1133 ± 4.24 ^a^	10.07 ± 0.08 ^a^	45.67 ± 0.18 ^a^	1.06 ± 0.01 ^a^	4.23 ± 0.01 ^a^	1.33 ± 0.01 ^a^	1.81 ± 0.01 ^a^	0.92 ± 0.00 ^a^	3.62 ± 0.06 ^a^	521 ± 3.54 ^a^	34.68 ± 0.18 ^a^	1.74 ± 0.03 ^a^
11%	244 ± 1.41 ^b^	1029 ± 9.19 ^b^	9.12 ± 0.16 ^b^	39.56 ± 0.70 ^b^	0.71 ± 0.01 ^b^	3.60 ± 0.05 ^b^	1.13 ± 0.02 ^b^	1.55 ± 0.02 ^b^	0.77 ± 0.01 ^b^	8.53 ± 0.56 ^b^	525 ± 2.12 ^b^	37.31 ± 0.28 ^b^	1.90 ± 0.03 ^b^
21%	223 ± 0.71 ^c^	935 ± 1.41 ^c^	8.19 ± 0.05 ^c^	32.78 ± 0.24^c^	0.73 ± 0.00 ^b^	3.31 ± 0.06 ^b^	1.04 ± 0.01 ^c^	1.43 ± 0.02 ^c^	0.70 ± 0.01 ^b^	14.53 ± 0.06 ^c^	494 ± 0.71 ^c^	39.57 ± 0.21 ^c^	1.64 ± 0.01 ^c^
31%	209 ± 0.00 ^d^	876 ± 0.00 ^d^	7.60 ± 0.06 ^d^	28.82 ± 0.23 ^d^	0.39 ± 0.01 ^c^	2.81 ± 0.03 ^c^	0.88 ± 0.01 ^d^	1.20 ± 0.01 ^d^	0.61 ± 0.01 ^c^	19.19 ± 0.22 ^d^	533 ± 1.41 ^b^	39.50 ± 0.11 ^c^	2.10 ± 0.03 ^d^

Data are expressed as mean ± SD. Means with different letters within each column are significantly different (*p* < 0.05).

**Table 6:** Gluten content of control bread, and 11%, 21% and 31% of BBH fortified breads.

Bread Type	Gluten (Ppm Per Loaf)	Gluten (mg/50 g Serving Size)	% Depletion in Gluten Per Loaf
Control	18903.00 ± 11.00 ^a^	6246.00 ± 3.60 ^a^	0.00
11%	17420.00 ± 107.00 ^a^	5712.00 ± 35.11 ^a^	−8.00
21%	13107.00 ± 523.00 ^b^	4229.00 ± 168.76 ^b^	−31.00
31%	7259.00 ± 748.00 ^c^	2335.00 ± 240.51 ^c^	−62.00

Data are expressed as mean ± SD. Means with different letters in the same row are significantly different (*p* < 0.05).
